# Indocyanine Green Fluorescence Angiography During Laparoscopic Bariatric Surgery: A Pilot Study

**DOI:** 10.3389/fsurg.2022.906133

**Published:** 2022-05-26

**Authors:** Andrea Balla, Diletta Corallino, Silvia Quaresima, Livia Palmieri, Francesca Meoli, Ingrid Cordova Herencia, Alessandro M. Paganini

**Affiliations:** Department of General Surgery and Surgical Specialties “Paride Stefanini”, Sapienza University of Rome, Rome, Italy

**Keywords:** indocyanine green (ICG), fluorescence angiography (FA), laparoscopic sleeve gastrectomy (LSG), laparoscopic gastric by-pass (LGB), bariatric surgery, leakage

## Abstract

**Aims:**

Indocyanine green (ICG) fluorescence angiography (FA) is used for several purposes in general surgery, but its use in bariatric surgery is still debated. The objective of the present pilot study is to evaluate the intraoperative utility of ICG-FA during bariatric surgery in order to focus future research on a reliable tool to reduce the postoperative leak rate.

**Methods:**

Thirteen patients (4 men, 30.8%, 9 women, 69.2%) with median age of 52 years (confidence interval, CI, 95% 46.2–58.7 years) and preoperative median body mass index of 42.6 kg/m^2^ (CI, 95% 36 to 49.3 kg/m^2^) underwent bariatric surgery with ICG-FA in our center. Three mL of ICG diluted with 10 cc sterile water were intravenously injected after gastric tube creation during laparoscopic sleeve gastrectomy (LSG) and after the gastric pouch and gastro-jejunal anastomosis creation during laparoscopic gastric by-pass (LGB). For the ICG-FA, Karl Storz Image 1S D-Light system (Karl Storz Endoscope GmbH & C. K., Tuttlingen, Germany) placed at a fixed distance of 5 cm from the structures of interest and zoomed vision modality were used to identify the vascular supply. The perfusion pattern was assessed by the surgical team according to a score. The score ranged from 1 (poor vascularization) to 5 (excellent vascularization) based on the intensity and timing of fluorescence of the vascularized structures.

**Results:**

Fom January 2021 to February 2022, six patients underwent LSG (46.2%), three patients underwent LGB (23.1%), and four patients underwent re-do LGB after LSG (30.8%). No adverse effects to ICG were observed. In 11 patients (84.6%) ICG-FA score was 5. During two laparoscopic re-do LGB, the vascular supply was not satisfactory (score 2/5) and the surgical strategy was changed based on ICG-FA (15.4%). At a median follow-up of five months postoperatively, leaks did not occur in any case.

**Conclusions:**

ICG-FA during bariatric surgery is a safe, feasible and promising procedure. It could help to reduce the ischemic leak rate, even if standardization of the procedure and objective fluorescence quantification are still missing. Further prospective studies with a larger sample of patients are required to draw definitive conclusions.

## Introduction

Bariatric surgery has strong evidence of efficacy and safety in the management of obesity and related comorbidities (1, 2). Laparoscopic sleeve gastrectomy (LSG) and laparoscopic gastric bypass (LGB) are among the most commonly performed bariatric procedures worldwide (1–4). Re-do laparoscopic gastric bypass (RLGB) proved to be an effective conversional procedure after LSG, in terms of total body weight loss, body mass index (BMI) loss and high remission rates of comorbidities (5).

Staple line dehiscence and leak after LSG and of the gastric pouch or gastro-jejunal anastomosis after LGB are the most severe and feared complications after bariatric surgery (6, 7), with an incidence rate ranging from 0 to 7% and from 0.1 to 8.3% for LSG and LGB, respectively (8, 9). The leak etiology is multifactorial with a sharing of mechanical and ischemic causes which has not been fully clarified yet (10, 11). In the literature, the mortality rate related to unrecognized leaks reaches 17% (7, 12). Leaks are associated with increased length of hospital stay and greater readmission rate leading to increased costs (13). The cumulative cost for complicated postoperative leak after bariatric surgery can exceed US $ 200,000 (7, 14, 15).

Several techniques have been described to prevent and to decrease the ischemic and mechanical leak incidence, including manual oversewing of the staple line or the use of sealants, or the use of staple line buttressing material (Seam-guard, Gore & Associates, Inc., Newark, Delaware, USA) (16, 17). However, the effectiveness of these techniques for leak prevention is still debated (9, 17–20). Moreover, various intraoperative leak detection modalities have also been described, such as the methylene blue test and intraoperative gastroscopy with air insufflation and hydropneumatic test, but the results are not univocal (21).

Some authors have recently used the intravenous administration of indocyanine green (ICG) to evaluate the real-time tissue perfusion during LSG, without achieving definitive results (22–24). ICG is a water-soluble anionic dye with hepatic excretion through the first pass effect (25, 26). It is safe since adverse events are reported in less than 1 in 40.000 patients and mostly including hypersensitivity reactions (25, 26). It is used in surgery for various purposes: evaluation of the anastomotic blood supply, visualization of the biliary tract during cholecystectomy, identification of the sentinel lymph node in breast cancer, melanoma and gastric carcinoma (27–32), but its use in bariatric surgery is still debated (22–24).

The objective of the present pilot study is to evaluate the intraoperative utility of ICG-FA during bariatric surgery in order to focus future research on a reliable tool to reduce the postoperative leak rate.

## Materials and Methods

This is a prospective observational pilot study. Institutional review board approval (RP120172B794FE16) and informed consent from all participants were obtained.

Fom January 2021 to February 2022, thirteen consecutive patients underwent bariatric surgery with ICG-FA in our center (Department of General Surgery and Surgical Specialties “Paride Stefanini”, Sapienza University of Rome, Rome, Italy).

### Preoperative Workup

All patients were candidate for bariatric surgery according to the Italian Society for Obesity and Metabolic Surgery (SICOB) guidelines as well as the European Association for Endoscopic Surgery (EAES) guidelines, as previously reported (33, 34).

Preoperative endoscopy with biopsies for Helicobacter Pylori (HP) detection was performed routinely (34–36). In case of HP infection, antibiotic therapy followed by preoperative urea breath test to prove its eradication, were performed (37).

The presence of signs of gastroesophageal reflux disease (GERD) was evaluated by endoscopy and patients’ symptoms were investigated by the Modified Italian Gastroesophageal reflux disease - Health-Related Quality of Life (MI-GERD-HRQL) questionnaire in all patients (38). When present at endoscopy, esophagitis was classified according to the Los Angeles Classification (39). Hiatal hernia (HH) was considered by calculating the distance between the lower edge of the palisade vessels and the diaphragmatic hiatus (40), but the definitive diagnosis was ascertained by intraoperative direct visualization of the esophageal hiatus.

Routine preoperative manometry, pH-metry and Rx-esophagogram were performed in accordance with Severe Acute Respiratory Syndrome CoronaVirus 2 (SARS-CoV-2) restrictions and patient compliance (41, 42). A De Meester score greater than 14.7 at pH-metry was considered abnormal.

Based on this preoperative workup, after multidisciplinary evaluation and after obtaining the patients’ informed consent, patients without GERD underwent LSG and patients with GERD underwent LGB. Patients with GERD after LSG, with or without weight regain, underwent RLGB.

In any case, if a diagnosis of HH was made intraoperatively, this was repaired with hiatoplasty according to guidelines (43), with mesh reinforcement as previously reported (36, 41).

### Surgical Techniques

Surgery was performed by the same surgeon (A.M.P.) and surgical team.

LSG was performed with a standardized technique as previously reported (34–36). Double loop technique was employed to perform both LGB and RLGB (44, 45). The bougie size was 36 F in all patients.

After gastric tube creation during LSG and after gastric pouch creation and gastro-jejunal anastomosis during LGB and RLGB, three mL of ICG (Pulsion Medical Systems SE, Feldkirchen, Germany) diluted with 10 cc of sterile water were injected intravenously. For the ICG-FA, Karl Storz Image 1S D-Light system (Karl Storz Endoscope GmbH & C. K., Tuttlingen, Germany) placed at a fixed distance of five centimeters from the structures of interest was used to identify the vascular supply.

During LSG, the rationale to ICG administration after gastric tube creation is related to the fact that to obtain sufficient weight loss, the stomach must be divided on the guide of the bougie. So ICG-FA is useful to decide the site of a manual stitch on the section line, if ischemic.

**Figure 1:**
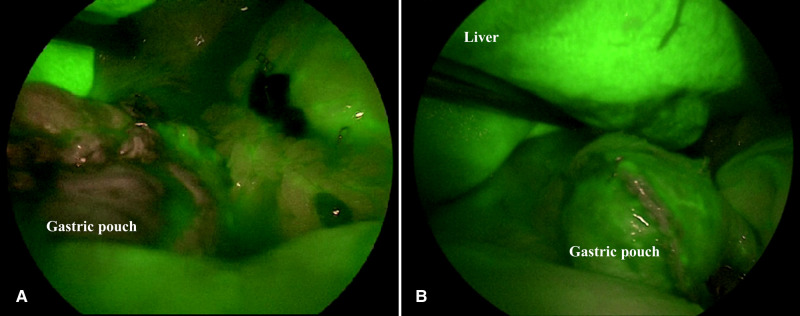
Intraoperative satisfactory ICG-FA score. ICG-FA: Indocyanine green-fluorescence angiography. (**A**) not perfused gastric pouch. (**B**) perfused gastric pouch.

The perfusion pattern was evaluated by the surgical team in real time, immediately after ICG injection and was assessed according to a score ranging from 1 (poor vascularization) to 5 (excellent vascularization) based on the intensity and rapidity of fluorescence appearance in the observed structures (Figure 1).

A score of 1–2 was considered inadequate and led to a change in surgical strategy, while a score of 3–5 was considered satisfactory, and the surgical strategy did not change. A methylene blue test is routinely performed after ICG-FA during LGB and RLGB. A routine Rx-esophagogram is performed on the third postoperative day after LGB and RLGB.

### Study Design

Preoperative variables (gender, age, BMI, associated comorbidities, smoking habit, American Society of Anesthesiologists - ASA - class, data obtained from endoscopy, Rx-esophagogram, MI-GERD-HRQL questionnaire, manometry, pH-metry and HP eradication), intraoperative variables (type of surgery, associated procedures, IGF-FA score, methylene blue test outcome, conversion to open surgery and operative time) and postoperative variables (complications according to Clavien-Dindo classification (46), postoperative hospital stay, mortality, follow-up data), were recorded using the Microsoft Excel program (Microsoft Corporation, Redmond, Washington, USA).

Continuous variables are expressed as median and 95% confidence interval (CI) while categorical variables are expressed as frequencies and percentages.

## Results

Tables 1 and 2 show patients’ characteristics and surgical details, respectively.

**Table 1:** Patients’ characteristics.

	Entire series, *N* = 13	LSG, *n* = 6(46.2%)	LGB, *n* = 3(23.1%)	RLGB, *n* = 4(30.8%)
Sex ratio (men : women)	4:9	3:3	0:3	1:3
Median age, years (CI 95%)	52(46.2–58.7)	51.5(37.3–57)	57(28.9–89.1)	55(42.5–68.5)
Median preoperative BMI, kg/m^2^ (CI 95%)	42.6(36–49.3)	52.6(39.3–59.9)	38.5(20–57.9)	35.6(19.8–50)
Comorbidities, *n*(%)				
T2DM	3(23.1)	2(33.3)	1(33.3)	–
Hypertension	7(53.9)	5(83.3)	2(66.7)	–
Sleep apnea syndrome^a^	8(61.5)	5(83.3)	2(66.7)	1(25)
Symptomatic GERD	7(53.9)	1(16.7)	3(100)	3(75)
Smoking habit	1(7.7)	–	–	1(25)
ASA class, *n*(%)				
II	5(38.5)	3(50)	–	2(50)
III	8(61.5)	3(50)	3(100)	2(50)
Endoscopic findings, *n*(%)				
Incontinent cardias	5(38.5)	1(16.7)	2(66.7)	2(50)
Hiatal hernia	7(53.9)	3(50)	3(100)	1(25)
Esophagitis	4(30.8)	2(33.3)	1(33.3)	1(25)
Gastritis	7(53.9)	4(66.7)	–	3(75)
Eradicated Helicobacter pylori, *n*(%)	1(7.7)	1(16.7)	–	–

*LSG, laparoscopic sleeve gastrectomy; LGB, laparoscopic gastric by-pass; RLGB, re-do laparoscopic gastric bypass; CI, confidence interval; BMI, body mass index; T2DM, Type 2 diabetes mellitus; GERD, Gastroesophageal reflux disease; ASA, American Society of Anesthesiologists.*

^a^ *Needing Continuous Positive Airway Pressure.*

**Table 2:** Surgical details.

	Entire series, *N* = 13	LSG, *n* = 6(46.2%)	LGB, *n* = 3(23.1%)	RLGB, *n* = 4(30.8%)
Associated procedures, n(%)	10(76.9)			
Hiatoplasty	5(38.5)	2(33.3)	2(66.7)	1(25)
Cholecystectomy	3(23.1)	2(33.3)	1(33.3)	–
Lysis of adhesions	1(7.7)	1(16.7)	–	–
Liver biopsy	1(7.7)	–	1(33.3)	–
ICG-FA score, n(%)	13(100)	6(100)	3(100)	4(100)
Satisfactory	11(84.6)	6(100)	3(100)	2(50)
Not satisfactory	2(15.4)	–	–	2(50)
Methylene blue test, n(%)	7(53.9)	–	3(100)	4(100)
Negative	7(53.9)	–	3(100)	4(100)
Positive	–	–	–	–
Conversion, n(%)	–	–	–	–
Median operative time, minutes (CI 95%)	285(193.6–294.1)	167.5(124.3–204.1)	320(226.3–427)	300(278.4–324.1)
Postoperative complications,				
*n*(%, Clavien-Dindo classification, grade)	3(23.1)	1(16.7)	1(33.3)	1(25)
Fever	2(15.4, II)	–	1(33.3)	1(25)
Wound infection	1(7.7, II)	1(16.7)	–	–
Median postoperative hospital stay, days (CI 95%)	4(3.3–5.5)	4(2.6–4.6)	5(−0.6–12.6)	4(1.9–6.6)
Mortality, *n*(%)	–	–	–	–
Median follow-up, months (CI 95%)	5(3.9–8.2)	5(2.3–8.4)	10(0.04–18)	5(−1.6–11.6)

*LSG, laparoscopic sleeve gastrectomy; LGB, laparoscopic gastric bypass; RLGB, laparoscopic re- gastric bypass; ICG-FA, Indocyanine green-fluorescence angiography; CI, confidence interval.*

Six (46.2%), three (23.1%) and four (30.8%) patients underwent LSG, LGB and RLGB, respectively. Ten patients underwent associated procedures (76.9%): hiatoplasty in 5 cases (38.5%), cholecystectomy in 3 (23.1%), lysis of adhesions from previous abdominal surgery in 1 (7.7%) and liver biopsy for severe hepatic steatosis in 1 (7.7%). All patients underwent preoperative multidisciplinary surgical-hepatological-radiological evaluation.

ICG-FA was performed in all patients with no adverse events, and without significant increase in operative time. In 11 patients ICG-FA score was 5/5 (84.6%). During two RLGB procedures, ICG-FA score was 2/5, and the surgical strategy was changed (15.4%).

In a 48-year-old woman with symptomatic GERD after LSG and weight regain (preoperative BMI 42.6 kg/m^2^), the ICG-FA demonstrated poor blood supply of a small area of the gastro-jejunal anastomosis (score 2/5), so reinforcement stitches were placed along the staple line.

In a 64-year-old woman with mild GERD and weight regain (preoperative BMI 43.5 kg/m^2^) after LSG the ICG-FA showed inadequate perfusion at the periphery of the gastric pouch (score 2/5). The gastric pouch was thus reshaped with the stapler, and the subsequent ICG-FA confirmed good perfusion of the residual gastric pouch (score 5/5).

Methylene blue test was negative in all cases. No conversion to open surgery occurred.

Postoperative complications were observed in three cases (23.1%): two patients with fever, and one with wound infection, all treated by antibiotic therapy (Clavien-Dindo grade II).

No leaks were observed at postoperative Rx -esophagogram. Median hospital stay was four days (CI, 95% 3.3–5.5 days) and mortality was nil. At a median follow-up of five months (CI, 95% 3.9–8.2 months) leaks did not occur.

## Discussion

The aim of this pilot study was to investigate if the intraoperative use of ICG-FA during bariatric surgery is a useful tool to reduce the postoperative ischemic leak rate. For this purpose, we prospectively analyzed the preliminary results of thirteen patients undergoing LSG or LGB with ICG-FA in our center. Even if leaks were not observed in any case, the use of ICG-FA changed the surgical strategy intraoperatively in two out of thirteen patients (15.4%), who underwent re-do surgery.

Leak of the staple line and of the gastric pouch or the gastro-jejunal anastomosis after LSG or LGB, respectively, are the most severe complications and the second most common cause of death after bariatric surgery (6, 7, 47). Knowing the exact pathogenesis would reduce the risk of a leak occurring after bariatric surgery. The leak etiology is multifactorial, and the causes fall into two main categories: mechanical/tissue causes and ischemic ones (10, 11, 48).

According to the mechanical theory, the intraluminal pressure due to pyloric conservation after LSG exceeds the strength of the staple-line, resulting in a leak (10). Mechanical leaks, which usually appear early after surgery (acute leaks), are usually related to the intrinsic characteristic of the long staple line (10, 49). The “weapons” described in the literature to prevent the onset of mechanical leaks have recently been re-evaluated (33). Routine staple line reinforcement (buttress, glues, suturing, clips) in LSG seems to reduce the risk of perioperative complications such as bleeding and overall mortality, but there does not appear to be a direct correlation between reinforcement and leak rate reduction (33, 50–52). Some authors describe the use of a bougie size ≥40 Fr as a leak-prevention technique (49, 53), but this has not been confirmed by the latest European Association for Endoscopic Surgery (EAES) guidelines which provide a conditional recommendation for the use of bougie sized ≤36 Fr (33, 54). Regarding the distance of the gastric transection from the pylorus, although some authors define 5–6 cm as a safety distance, this data are not confirmed by the most recent literature (49, 55–57).

On the other hand, according to the ischemic theory, leaks are due to localized ischemia which occurs most frequently in the “critical area” at the level of the angle of His after gastroepiploic and short gastric vessels ligation (10). Ischemic leaks, occurring after postoperative day 7 (early leaks), can be partially prevented by maintaining a safety distance of 1–2 cm from the gastroesophageal junction during LSG and they are also a major challenge during revisional bariatric surgery, in which tissues are more frequently hypo-perfused (10, 58, 59). In this situation, the use of ICG-FA could be useful to prevent leak.

Several modalities of intraoperative leak diagnosis (air leak test, intraoperative endoscopy, dye leak test) have been described, with discordant results (21, 53, 60). First of all, the intraoperative leak test might be useful to identify staple line disruption, but it does not allow to identify an ischemic area at risk for subsequent leak (24, 60). Furthermore, the stress of the intraoperative leak test on the newly formed staple line may itself be the cause of staple line weakness and leak (24, 53).

Some authors have recently used the intraluminal or intravenous administration of ICG to evaluate real-time tissue perfusion and to assess the integrity of the staple line during bariatric surgery, without, however, achieving definitive results yet (22–24, 27, 61).

In the literature, the use of ICG-FA during bariatric surgery is reported only in three articles (22–24), and by the results of Spota et al. which report data obtained from 129 bariatric procedures with ICG-FA retrieved from the European-Fluorescence Imaging-Guided Surgery (EURO-FIGS) registry (27). They reported that the choice of the anastomotic level was ICG-FA unrelated in almost all cases and that the ICG-FA was primarily used to assess blood perfusion of the anastomoses, with a partial or high surgeons’ sense of confidence (27).

Frattini et al. used ICG-FA in 15 patients undergoing LSG (23). They reported that ICG-FA was feasible and comparable with other intraoperative or postoperative tests (including methylene blue test and Rx-esophagogram) in terms of leak detection rate, and it also allowed for a real-time assessment of gastric perfusion (23). In our opinion the latter feature gives added value to ICG-FA. In fact, although the methylene blue test was negative, in the present series, in two cases of RLGB, the ICG-FA showed that the vascular supply was lower than 3/5, underlining how the two tests are complementary to each other, highlighting one the mechanical causes of the leak and the other the ischemic ones.

Di Furia et al. performed LSG with ICG-FA in 45 patients with the aim of clarifying the exact pathogenesis of gastric leak supporting the ischemic theory and to evaluate if ICG-FA could adequately estimate the ischemic area along the staple line and to prevent leaks (24). They defined adequate perfusion as “the direct and clear visualization of fluorescence along the gastric tube, compared with the excised specimen, after an estimated time of 150–180 seconds from intravenous injection”. Despite intraoperative methylene blue test and postoperative Rx-esophagogram were negative for leak in all patients and the ICG-FA score was evaluated “satisfactory and adequate” in all patients, one patient in their series developed symptomatic leak on the fifth post-operative day (24). Therefore, the leak detection rate of the various tests would seem to be comparable, but not reliable probably because the main causes of gastric leak arise not during the procedure or in the early postoperative course, but, as confirmed by the literature, later (5, 8). This data suggests the poor role of the ICG-FA, as well as other leak tests, during LSG probably because the ischemic theory alone is not able to explain the onset of the leakage, emphasizing once again its multifactorial etiology (8, 10, 11, 24).

In our opinion, although this conclusion can be applied to LSG, we do not believe it is entirely true for revisional bariatric surgery. To the best of our knowledge, this is the first study evaluating the role of ICG-FA during primary or revisional LGB. In this series the only two cases in which the ICG-FA determined a change in surgical strategy were RLGB, suggesting how the ICG could have a higher detection rate of ischemic areas during revisional surgery.

With the same goal, Ortega et al. performed ICG-FA before the gastric division during LSG to identify the variable blood supply patterns of the gastroesophageal junction and the ICG-FA was repeated at the end of the procedure to ensure that all the pertinent blood vessels were preserved (22). They have identified three patterns of blood supply: a right-side–dominant pattern (20%), arising from the left gastric artery; a right-side–accessory pattern (36%), running in the gastrohepatic ligament and a left-side accessory pattern (34%) arising from tributaries from the left inferior phrenic artery significantly contributing to the right-side blood supply (22). Therefore ICG-FA represents a good strategy to avoid unnecessary injury to these vessels during the procedure thanks to “perfusion-preserving” dissection and checking for adequate perfusion of the sleeve product afterward (22).

Finally, another use of ICG described in the literature is the intraluminal gastric injection (61). Kalmar et al. performed intraoperative leak test using the intraluminal ICG method in 59 patients and the gastroscopy method in 196 patients who underwent bariatric surgery, proving that intraluminal ICG is an alternative for intraoperative detection of leak with comparable specificity to intraoperative endoscopy (61). The advantage of the intraluminal ICG, in comparison to intraoperative endoscopy, is that it does not require an experienced endoscopist, any personnel and material cost to resterilize the equipment and not increasing the operative time (61). It is a similar test with a reported lower false negative rate than the methylene blue test, although there are no comparative studies in the literature (21, 61, 62). This ICG using modality is different from the one employed in our experience, and it is aimed at identifying the mechanical rather than vascular causes of the onset of leaks (61). It may be useful to evaluate the feasibility of concomitant administration of ICG both orally and intravenously.

No adverse effects of ICG administration were observed in the reported series as in the present analysis, which therefore is confirmed to be a safe dye (22, 24, 27).

In the literature a wide heterogeneity regarding the doses and timing of ICG administration is reported (25), as well as the distance of the laparoscope from the target organs during visualization, and finally the mostly subjective evaluation of the vascularity which does not allow to make the interpretation of fluorescence intensity objective. Although we have tried to standardize our technique, through the administration of a standard dose of ICG, at the same time and at a fixed optic distance and with an ICG-FA score evaluated by the same surgical team, there are still several limitations of the present study, such as the small sample size, the lack of a control group and the lack of a quantitative fluorescence evaluation. During the study period, our Institute was converted into a SARS-CoV-2 hospital, partially justifying the paucity of the study cohort. With the end of the state of emergency, we hope to increase the number of bariatric procedures with ICG-FA, aiming to enlarge the patients sample size and to draw more definitive conclusions.

In the present study the use of ICG-FA allowed to identify an ischemic area leading to a change in the surgical strategy in half of the patients undergoing revisional bariatric surgery. Although this result could be influenced by the small sample size, this is not yet described in the literature, so if our preliminary results will be confirmed, the use of ICG-FA could represent a decisive weapon in the prevention of ischemic leak in revisional bariatric surgery.

In conclusion, based on the present study, ICG-FA during bariatric surgery is a safe and feasible procedure. Its use could reduce the ischemic leak rate, especially in patients undergoing revisional bariatric surgery even if standardization of the procedure and objective fluorescence quantification are still missing. Definitive data from this pilot study increasing the number of patients included, and further prospective studies with a larger number of patients are required to draw definitive conclusions.

## Data Availability

The raw data supporting the conclusions of this article will be made available by the authors, without undue reservation.
